# Toll-Like Receptors and Cancer, Particularly Oral Squamous Cell Carcinoma

**DOI:** 10.3389/fimmu.2014.00464

**Published:** 2014-09-24

**Authors:** Alison Mary Rich, Haizal Mohd Hussaini, Venkata P. B. Parachuru, Gregory J. Seymour

**Affiliations:** ^1^Faculty of Dentistry, Sir John Walsh Research Institute, University of Otago, Dunedin, New Zealand; ^2^Faculty of Dentistry, Department of Oral Pathology and Oral Medicine, National University of Malaysia, Kuala Lumpur, Malaysia

**Keywords:** toll-like receptors, TLR, NF-κB, oral squamous cell carcinoma

## Abstract

It is becoming increasingly apparent that the tumor microenvironment plays an important role in the progression of cancer. The microenvironment may promote tumor cell survival and proliferation or, alternatively may induce tumor cell apoptosis. Toll-like receptors (TLRs) are transmembrane proteins, expressed on immune cells and epithelial cells, that recognize exogenous and endogenous macromolecules. Once activated, they initiate signaling pathways leading to the release of cytokines and chemokines, which recruit immune cells inducing further cytokine production, the production of angiogenic mediators and growth factors, all of which may influence tumor progression. This paper examines the actions of TLRs in carcinogenesis with particular emphasis on their role in oral squamous cell carcinoma.

## Introduction

Toll-like receptors (TLRs) are cell surface or intracellular transmembrane proteins that are expressed on sentinel cells of the immune system such as macrophages and dendritic cells. In addition, they are present on non-immune cells such as keratinocytes of skin (1) and oral mucosa (2), gastrointestinal (3), and female reproductive tract lining (4). On these lining epithelia, TLRs act as sensors where they recognize pathogens and are activated when the epithelium is disturbed. Microbial pathogens are characterized by specific arrangements of molecules known as pathogen-associated molecular patterns (PAMPs), which can be recognized by pattern-recognition receptors (PRRs), including TLRs. TLRs are capable of recognizing bacterial, viral, fungal, and protozoal components. While their role as PRRs is important in host defense, it is their ability to regulate innate and adaptive immune responses *via* information from surface epithelial cells that is their most potent role, along with their ability to recruit immune cells (5).

Once activated, TLRs trigger co-ordinated expression of genes involved in specific signaling pathways in the regulation of innate and adaptive immunity and tissue repair and regeneration. Their cytoplasmic domain has extensive homology with the interleukin (IL)-1 receptor family and is known as the Toll-IL receptor (TIR) domain (6). With binding of ligand to TLRs, there is activation of signaling transduction pathways involving TIR with coupling to adaptor molecules including myeloid differentiation factor 88 (MyD88), TIR domain-containing protein (TIRAP), and TIR domain-containing adaptor inducing interferon-β-related adaptor molecule (TRAM). This potentially leads to the activation of two main pathways, the MyD88-dependent (used by all TLRs except TLR3) and the MyD88-independent TRAM/TRIF pathway (used by TLR3 and some signals of TLR4) (5). Signaling through the MyD88 pathway leads to activation and translocation from the cytoplasm to the nucleus of the transcription factor nuclear factor-κB (NF-κB). There it binds to the promoter region of a variety of immune and inflammatory genes leading to the transcription of inflammatory and anti-inflammatory cytokine genes, e.g., tumor necrosis factor (TNF)-α and IL-6 (3, 4, 7). Activation of the TRAM/TRIF pathway leads to the production of type 1 interferons. In this manner, TLRs regulate the production of cytokines, opsonization, coagulation cascades, complement activation, and upregulation of co-stimulatory molecules on antigen presenting cells (4, 8, 9). Alteration to TLR genes, as occurs with single-nucleotide polymorphisms (SNPs), may interfere with the function of TLRs and shift the balance of the cytokines produced (10). A further important function of TLRs is the induction of apoptosis through the expression of anti-apoptotic proteins and apoptosis inhibitors (11).

Initially, study of TLRs in pathology was concentrated on their association with microbial pathogens. It is increasingly apparent that TLRs also recognize damage/danger-associated molecular patterns (DAMPs), endogenous molecules released from damaged and dying cells. DAMPs include heat shock proteins (HSP), nucleic acids, fibrinogen, and high-motility group box-1 protein (HMGB1) (3, 12, 13). DAMPs can be released from cells that have been affected by various stimuli and have entered a potentially neoplastic phase, as well as from cells that have undergone malignant transformation (14, 15). This has led to a large number of studies investigating the role of TLRs in the pathogenesis of a range of malignant neoplasms. The association of TLRs with neoplasia will be discussed below, with particular emphasis on two points: (a) how this information can be used to advance our knowledge of the association between TLRs, inflammation, and cancer, particularly oral squamous cell carcinoma (OSCC) and (b) how it can be used to develop new therapeutic strategies.

## Inflammation, TLRs, and Cancer

Infection is an important cause of cancer, causing approximately one in five malignancies worldwide (16, 17). Infection with the bacterium *Helicobacter pylori* leads to an elevated risk of developing gastric adenocarcinoma and gastric lymphoma, infection with particular types of human papilloma virus (HPV) leads to cervical cancer, tonsillar carcinoma, and some cases of OSCC, and chronic hepatitis B and C infections leads to hepatocellular carcinoma (16–19). The herpesvirus, Epstein–Barr virus (EBV), is implicated in a range of malignancies including Burkitt’s lymphoma, Hodgkin lymphoma, and nasopharyngeal carcinoma, and another member of the herpesvirus family, human herpesvirus 8, is a causal factor in Kaposi sarcoma (17, 18). The response of TLRs to these infections is crucial to the evolution of the infection and possibly to the transformation to malignancy, but a full review of infection and cancer is beyond the scope of this review. Instead, we intend to concentrate on the role of TLRs in tumor development, whether or not the tumor was associated with prior infection. The modulation of the inflammatory process by TLRs is a key factor in tumor development and progression, inducing both tumor-promoting and anti-tumor responses (15, 20). TLRs have also been shown to play a crucial role in tissue repair and regeneration following injury, particularly in relation to epithelial regeneration and myofibroblast activation (20–22) These processes may be mediated by TLRs providing pro-survival signals and by preventing apoptosis and hence may dictate the balance between satisfactory and maladaptive wound healing (21, 22).

**Figure 1:**
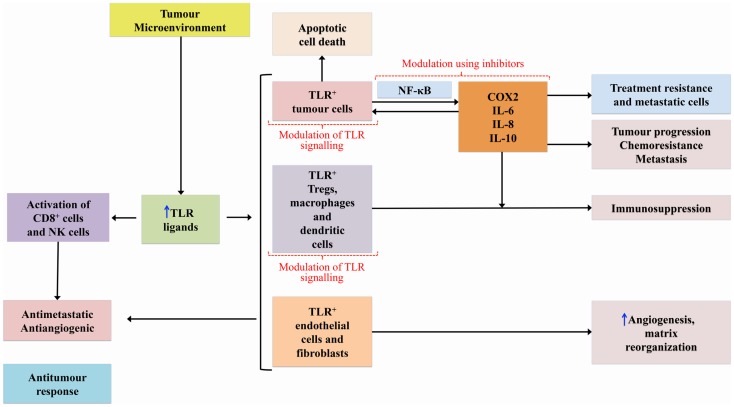
**Diagram showing how TLRs can mediate pro- and anti-tumor pathways**.

Genetic disruption of TLRs and adaptor molecules of the TLR pathway has been associated with tumor development and progression in mice (23, 24). TLRs mediate both pro- and anti-tumorogenic pathways, the so-called double-edged sword (25, 26) – see Figure 1. DAMP activation of TLRs expressed on tumor cells initiates cascades that mediate the release of cytokines and chemokines from the tumor cells which, in turn, recruit immune cells leading to upregulation of NF-κB signaling and subsequent release of additional cytokines, pro-angiogenic mediators, growth factors, and anti-apoptotic proteins that continue to promote tumor survival and progression (15, 20, 27). It should be noted, however, that the role TLRs play in relation to apoptosis is complex and variable with evidence that they can prevent apoptosis, but also have pro-apoptotic activity through a range of mechanisms (28, 29).

The nuclear transcriptional factor NF-κB is constitutively expressed in many human cancers (30, 31), and its signaling pathway has a critical role in carcinogenesis (32, 33). Mutations in NF-κB signaling molecules have been reported in a range of malignancies, including human B-cell lymphomas (34). NF-κB is likely to be linked to this process through its ability to induce expression of adhesion molecules, MMPs, and pro-inflammatory cytokines, which creates a microenvironment favorable for cancer cell survival (7, 32). A potential feedback loop between pro-inflammatory cytokines such as TNF-α and NF-κB has been suggested and this links the role of NF-κB in inflammation and cancer (32, 35). It has also been suggested that NF-κB contributes to genomic instability through its anti-apoptotic activities, in addition to the suppression of apoptosis in tumor cells, which promotes tumor cell survival (36, 37). The NF-κB signaling pathway is an essential anti-apoptotic pathway that has been shown to control the expression of anti-apoptotic genes and restrict the activation of pro-apoptotic pathways (20).

It has been suggested that the amplitude and duration of TLR activation may be instrumental in tumor development, with chronic low-grade activation favoring a tumor-promoting pro-inflammatory state and with high dose therapeutically induced TLR activation favoring an anti-tumor response (20). Various TLRs play different roles in carcinogenesis. TLR4 shows pro-tumorogenic effects, with mice deficient in TLR4 at reduced risk of developing gastrointestinal and hepatocellular cancer (23, 38). This effect has been shown to be due to augmentation of the inflammatory response *via* activation of the NF-κB, and cyclooxygenase (Cox)-2/prostaglandin E2 signaling pathways (23, 38). The TLR4 pro-tumorogenic activity is mainly due to its expression on tumor cells where it mediates resistance of tumor cells to damage induced by cytotoxic T lymphocytes (25, 37), but triggering of TLR4 expressed on immune cells is also important in tumor development and progression (37). Release of HSP from tumor cells can lead to activation of TLR4 on tumor-associated macrophages, in turn mediating NF-κB activation of tumor cells (39). While TLR4 is predominately pro-tumorogenic, it can induce the production of interferons contributing to an anti-tumor response (37).

Both TLR3 and TLR5 are also pro-tumorogenic with their signals mediating tumor invasion and metastasis by enhancing cell migration, but, like TLR4, also have an anti-cancer effect in some situations (14, 40, 41). TLR9 expressed on tumor cells has been associated with increased cell proliferation and increased invasion potential (42, 43). On the other hand, TLR2 signaling suppressed cancer development and assisted tumor regression (37). Lack of TLR2 led to increased cell proliferation and decreased apoptosis in a mouse model of colorectal cancer (24). The mechanism by which TLR2 induces tumor suppression is thought to be mediated through tumor-derived HMGB1, which activates TLR2 in dendritic cells in the tumor microenvironment, leading to tumor regression (44). While most reports describe the anti-tumor functions of TLR2, opposing results have been reported in a context-dependent manner with TLR2 playing an important tumor-promoting role in gastric cancer (20, 45, 46).

## Association of TLRs with Oral Cancer

**Figure 2:**
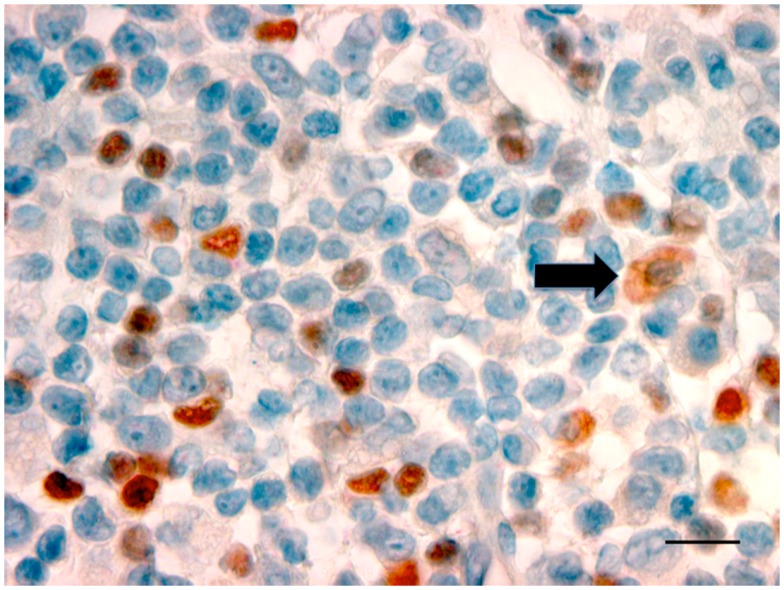
**Photomicrograph showing TLR2^+^ cells with a dusky red cell membrane and cytoplasmic staining (black arrow) with the morphology of macrophages in OSCC (ȕ100, bar: 100 μm)**.

Oral and pharyngeal cancer is the sixth most common cancer in the world, with oral cancer having an annual estimated incidence of approximately 275,000 globally and with two-thirds of cases occurring in developing countries (47). Despite advances in diagnosis and improved treatment strategies, OSCC continues to have a poor 5-year survival rate, and conventional treatment modalities of surgery and radiotherapy are associated with significant morbidity (48). OSCC, like other cancers, develops in an immune cell-rich environment, where inflammatory cells in the tumor microenvironment establish an anti-tumor response by secreting pro-inflammatory cytokines. At the same time, the cancer cells may induce various mechanisms suppressing the anti-tumor response, such as regulating a network of suppressive cytokines and the recruitment of suppressive regulatory T-cells (Tregs). Studies have shown TLRs, particularly TLR2, play a role in Treg expansion and their suppressive capacity (49). More keratinocytes in OSCC expressed TLR2 than keratinocytes in control epithelium (50). Further research in our laboratory has confirmed the presence of TLR2^+^ cells within the OSCC microenvironment (51). The cells that were expressing TLR2 were large and morphologically consistent with macrophages or DC (Figure 2). The presence of TLR2^+^FoxP3^+^ cells within the OSCC immune cell infiltrate was noted, an observation previously unreported in OSCC. This is important, particularly in relation to cancer immumotherapy. Cells expressing TLRs could potentially be modulated to shift the environment toward a Th1 environment, thus stimulating the pro-inflammatory process.

Head and neck cancer cell lines and OSCC tissue specimens were found to express TLR3, and this was associated with high levels of expression and activity of NF-κB (40, 41, 52). Inhibition of TLR3 by siRNA resulted in decreased expression of the NF-κB-regulated oncogene c-myc and decreased cell proliferation (53). Stimulation of TLR3^+^ OSCC tumor cells promoted their migration, and TLR3 expression was found to be significantly correlated with poor differentiation and perineural invasion in OSCC (40). Further, activation of TLR3 was found to induce apoptosis in OSCC cells, most likely mediated by activation of the IRF/IFN-β signaling pathway (41).

TLR4 and MyD88 were expressed in human OSCC cell lines and expression level correlated with tumor differentiation, with higher expression in more well-differentiated carcinomas (54). Expression of TLR4 increased with increasing degrees of oral epithelial dysplasia (55) and distribution of TLR4 extended from the basal layer through the *stratum spinosum* as dysplasia progressed.

TLR5 expression was more pronounced in tongue cancer cells than in adjacent apparently normal epithelium and high expression levels of TLR5 predicted a poorer prognosis (14). While, overall, there was relatively little TLR9 expression in dysplastic oral tissue, there was significantly more than in normal oral mucosa (55) and increased expression of TLR9 had earlier been found to correlate with increased tumor cell proliferation in OSCC (56, 57). Subsequent reports suggested that TLR9 directly promoted cancer cell invasion (58).

## Therapeutic Possibilities

Since advances in conventional treatments have not appreciably increased the survival rate of OSCC (48), attention has turned to immunotherapy including investigation of drugs that interfere with the NF-κB and TLR signaling pathways. Interference with these pathways has implications for many of the important steps in carcinogenesis including the relationship between TLR^+^ cells and regulatory T-cells, and subsequent cytokine production, the effects on dendritic cells and macrophages, the effects on apoptosis and on cell proliferation. Acetylsalicylic acid has been shown to reduce the long-term risk of developing some cancers, and, while the precise mechanisms of action remain uncertain, there is evidence that modulation of the NF-κB signal transduction pathway by aspirin and other non-steroidal anti-inflammatory agents is an important mechanism in inducing apoptosis in neoplastic epithelial cells (59, 60). Meyer et al. (33) found aspirin and another COX-2 inhibitor, celecoxib inhibited NF-κB in head and neck cancer cell lines. Both selective and non-selective COX-2 inhibitors reduced cancer incidence and invasion score in an animal model of experimental oral carcinogenesis, although the potential mechanisms of actions of the drugs were not discussed in detail (61). Drugs other than COX inhibitors have been shown to interfere with the NF-κB pathway in various malignancies, e.g., glucocorticoids, while being pro-apoptotic in leukemia and lymphomas, have been found to enhance survival of some breast cancer cell lines through promoting NF-κB transcriptional activity leading to upregulation of c-*Myc* and enhanced anti-apoptotic function (62, 63). Sulfasalazine can act as a NF-κB inhibitor leading to reduced proliferation and enhanced apoptosis of esophageal cancer cell lines (64) and has be shown to induce autophagic cell death in oral cancer cell lines, although this was thought to be mediated via Akt and ERK pathways (65).

It is likely that at least some of the effects on NF-κB are mediated through various TLR pathways. TLR4 induced resistance to cisplatin-induced apoptosis in OSCC cell lines (54). The authors suggested that activation of TLR4 and its signaling pathways was a mechanism to explain the resistance that develops in patients with OSCC, after a good initial response to that drug. The potential use of the immunostimulatory properties of TLR agonists, particularly TLR2, TLR3, TLR4, TLR7/8, and TLR9, has been investigated in cancer immunotherapy (66). Sutmuller and colleagues (49) showed that TLR2 controls the expansion and suppression of Tregs by directly acting on these cells. They also showed that there was a temporary loss of Treg suppressive function when bound to a TLR2 ligand; concurrently, this could also induce the proliferation of CD4^+^CD25^+^ Tregs. Subsequently, it was shown that TLR2 agonists enhanced the suppressive effect of Tregs (67). This observation may explain in part the failure of some immunotherapies, in particular, the use of TLR agonists as anti-cancer treatment. By inducing apoptosis, necrosis, and activating immune and dendritic cells, TLR agonists induce an anti-tumor effect (68). Topical imiquimod, a synthetic TLR7 agonist, that has been shown to induce temporary regression of breast cancer in an animal model (69) and has had some success in the management of varying types of dysplastic skin lesions and superficial skin cancers (70, 71). It has been shown to reduce the degree of dysplasia in a mouse model of oral cancer (although there was no long-term follow-up) (72) and in OSCC cell lines (73).

Although much anticipated, the outcome of TLR agonist therapeutic trials has been disappointing (74). Clinical studies with TLR agonists have shown expansion of Tregs within the tumor environment, which may enhance tumor progression, although some trials have shown reduction in tumor size (69, 75). This has earned TLRs the connotation of a “double-edged sword” by most workers who investigated their functionality (76, 77). Hence, the therapeutic use of TLR agonists needs to be undertaken with caution. TLR antagonists are structural analogs of agonists, which bind to TLRs and do not induce signal transduction. This prevents the agonistic action of TLR ligands responsible for the induction of inflammatory responses (78, 79). These agents can be used to gain a greater understanding of the effectiveness of TLR agonist therapy in animal models of cancer.

To overcome the “double-edged sword,” manipulation of the “un-touched” TLR^+^ cells within the OSCC tumor microenvironment is required. The key factors lie in the balance of the cytokine network within the tumor environment. It is crucial to look into the expression of various cytokines at different stages of OSCC.

Inhibition of NF-κB in head and neck cancer cell lines was associated with downregulation of TLR3 and decreased levels of IL-6 and IL-8 and the expected shift to a T_H_2 microenvironment, typical in head and neck squamous cell carcinomas did not occur (33). Modulation of macrophages or DCs using the cytokine network is necessary to overcome potential immune suppression, before they are recruited to become tolerogenic cells. The administration of agents that interfered with TLR3 signaling in a mouse model of lung cancer led to tumor regression by converting tumor-supporting macrophages to tumor suppressors (80). Again, the presence of “un-touched” TLR^+^ macrophages supports the theory that reversal or “resurgence” by the immune system is important in fighting back against the tumor escape mechanisms.

The association of TLRs with tumor cell proliferation is another potential area for therapeutic intervention. TLR3 (52) and TLR9 (56) have been associated with cell proliferation in head and neck cancer, and Ruan et al. (58) suggested that the use of anti-TLR9 agents may reduce tumor cell proliferation in OSCC as well as reducing metastatic potential.

Although complete molecular mechanisms remain to be determined, there are a variety of TLR pathways that may provide therapeutic targets for the management of OSCC.

## Conflict of Interest Statement

The authors declare that the research was conducted in the absence of any commercial or financial relationships that could be construed as a potential conflict of interest.
